# MRI-based machine learning models predict the malignant biological behavior of meningioma

**DOI:** 10.1186/s12880-023-01101-7

**Published:** 2023-09-27

**Authors:** Maoyuan Li, Luzhou Liu, Jie Qi, Ying Qiao, Hanrui Zeng, Wen Jiang, Rui Zhu, Fujian Chen, Huan Huang, Shaoping Wu

**Affiliations:** 1 Department of Radiology, Chengdu Qingbaijiang District People’s Hospital, Chengdu, 610300 Sichuan China; 2 Department of Radiology, The First Affiliated Hospital of Chengdu Medical College, Chengdu, 610500 Sichuan China; 3 Department of Radiology, Mianyang Central Hospital, Mianyang, 621000 Sichuan China; 4 Department of Radiology, The Affiliated Traditional Chinese Medicine Hospital of Southwest Medical University, Luzhou, 646000 Sichuan China; 5 Department of Radiology, Chengdu Medical College, Chengdu, 610500 Sichuan China; 6 Department of Radiology, Sichuan Taikang Hospital, Chengdu, 610041 Sichuan China

**Keywords:** Magnetic resonance imaging, Machine learning, Meningioma, WHO grade, Ki-67

## Abstract

**Background:**

The WHO grade and Ki-67 index are independent indices used to evaluate the malignant biological behavior of meningioma. This study aims to develop MRI-based machine learning models to predict the malignant biological behavior of meningioma from the perspective of the WHO grade, Ki-67 index, and their combination.

**Methods:**

This multicenter, retrospective study included 313 meningioma patients, of which 70 were classified as high-grade (WHO II/III) and 243 as low-grade (WHO I). The Ki-67 expression was classified into low-expression (n = 216) and high-expression (n = 97) groups with a threshold of 5%. Among them, there were 128 patients with malignant biological behavior whose WHO grade or Ki-67 index increased either or both. Data from Center A and B are were utilized for model development, while data from Center C and D were used for external validation. Radiomic features were extracted from the maximum cross-sectional area (2D) region of Interest (ROI) and the whole tumor volume (3D) ROI using different paraments from the T1, T2-weighted, and T1 contrast-enhanced sequences (T1CE), followed by five independent feature selections and eight classifiers. 240 prediction models were constructed to predict the WHO grade, Ki-67 index and their combination respectively. Models were evaluated by cross-validation in training set (n = 224). Suitable models were chosen by comparing the cross-validation (CV) area under the curves (AUC) and their relative standard deviations (RSD). Clinical and radiological features were collected and analyzed; meaningful features were combined with radiomic features to establish the clinical-radiological-radiomic (CRR) models. The receiver operating characteristic (ROC) analysis was used to evaluate those models in validation set. Radiomic models and CRR models were compared by Delong test.

**Results:**

1218 and 1781 radiomic features were extracted from 2D ROI and 3D ROI of each sequence. The selected grade, Ki-67 index and their combination radiomic models were T1CE-2D-LASSO-LR, T1CE-3D-LASSO-NB, and T1CE-2D-LASSO-LR, with cross-validated AUCs on the training set were 0.857, 0.798, and 0.888, the RSDs were 0.06, 0.09, and 0.05, the validation set AUCs were 0.829, 0.752, and 0.904, respectively. Heterogeneous enhancement was found to be associated with high grade and Ki-67 status, while surrounding invasion was associated with the high grade status, peritumoral edema and cerebrospinal fluid space surrounding tumor were correlated with the high Ki-67 status. The Delong test showed that these significant radiological features did not significantly improve the predictive performance. The AUCs for CRR models predicting grade, Ki-67 index, and their combination in the validation set were 0.821, 0.753, and 0.906, respectively.

**Conclusions:**

This study demonstrated that MRI-based machine learning models could effectively predict the grade, Ki-67 index of meningioma. Models considering these two indices might be valuable for improving the predictive sensitivity and comprehensiveness of prediction of malignant biological behavior of meningioma.

**Supplementary Information:**

The online version contains supplementary material available at 10.1186/s12880-023-01101-7.

## Introduction

Meningioma is the most common primary intracranial tumor, accounting for 37.6% of all central nervous system tumors [1]. The high-grade meningiomas (WHO grade II-III) account for approximately 20% of all types of meningiomas and are associated with a higher recurrence rate and poorer prognosis [2, 3]. Although most meningiomas are benign (WHO Grade I), their prognosis is not always favorable. The Ki-67 index is a histopathological marker, and its high expression typically indicates accelerated and uncontrolled cell proliferation that is correlated with tumor growth [4]. Accumulating evidence suggests that a high Ki-67 index is an independent prognostic predictor directly associated with an increased recurrent risk following surgical resection [5, 6]. It’s important to noninvasively predict the grade of meningioma patients and shorten the time of follow-up before surgery [7].

Studies have demonstrated that some MRI radiological features, such as heterogeneous enhancement and intratumoral necrosis, correlated with meningioma grade and Ki-67 index. Some studies have also suggested that the machine learning model based on radiomic was feasible for preoperative prediction of meningioma grade [8, 9] and Ki-67 index [10, 11]. However, there is no literature on classification for the grade and Ki-67 index alone simultaneously and prediction of biological behavior of meningioma from the perspective of their combination.

The aim of this study was to develop and validate MRI-based machine learning models to predict the malignant meningioma biological behavior from the perspective of the WHO grade, Ki-67 index, and the combination of both indices.

## Methods

### Patients

A total of 483 patients who underwent meningioma resection between January 1, 2016 and July 31, 2023 were initially collected from centers A(n = 339) and B(n = 46), C(n = 55), D(n = 43). Pathology reports were reviewed to ensure that they met the criteria of the 2021 WHO classification criteria for central nervous system tumors.

Patients meeting the following inclusion criteria were selected: [1] underwent MRI examination before surgery, [2] had a confirmed diagnosis of meningioma by pathology, and [3] did not receive any treatment prior to MRI and surgery. Patients were excluded if they met any of the following exclusion criteria: [1] unclear pathological diagnosis or missing grade or Ki-67 index information, [2] severe MRI image artifacts or poor image quality unsuitable for analysis, and [3] recurrence, multiple lesions, or lesions smaller than 1 cm×1 cm×1 cm. Clinical features, including age, sex, grade, and Ki-67 index were recorded for each patient.

Based on the above criteria, 313 patients in total (183 from the center A, 41 from the center B, 50 from the center C, and 39 from the center D) were included in the study. Data from Center A and B were employed for model development, while data from Center C and D were utilized for external validation.

### Image acquisition

MRI scans were conducted at four different centers using various scanners. 1.5T Siemens Avanto and 1.5T GE Signa HDxt scanners at centers B and C, respectively, and 3.0T Philips Achieva and 3.0T Siemens Skyra scanners at centers A and D, respectively. All patients underwent standard MRI examinations with a slice thickness was 5 mm. Contrast-enhanced MRI scans were acquired after injection of gadodiamide (dose: 0.1 mmol/kg) as a contrast agent. The dynamic-enhanced MRI scan was performed within 250 s of contrast injection. Detailed parameters can be found in the supplementary material.

### Image preprocessing and tumor segmentation

Raw MRI data of Digital Imaging and Communications in Medicine (DICOM) format were first converted to Neuroimaging Informatics Technology Initiative (NIfTI) format using ITK-SNAP (version 3.8.0, University of Pennsylvania, Philadelphia, USA; http://www.itksnap.org). Image preprocessing was required before radiomic feature extraction, including nonparametric nonuniform intensity normalization algorithm (N4ITK) bias correction, normalization at a scale of 100, resampling to a 1 × 1 × 1 mm^3^ resolution, and gray-level intensity normalization in the range of 0 to 255 [10, 11]. The ITK-SNAP software was also utilized for tumor segmentation. Blinded to patient information, two radiologists with more than five years of experience in image reading manually segment region of interest (ROI) for all data, including the largest cross-sectional area of the tumor (2D) ROI and the whole tumor (3D) ROI. A senior neuroradiologist with over ten years of experience in image reading reviewed and selected ROIs used to radiomic features extraction. Interobserver agreement was assessed by calculating the intraclass correlation coefficients (ICCs) between the two radiologists in training set. Only radiomic features with high ICCs (ICC ≥ 0.8) were included in the modeling process.

### Clinical and radiological features collection and selection

**Fig. 1:**
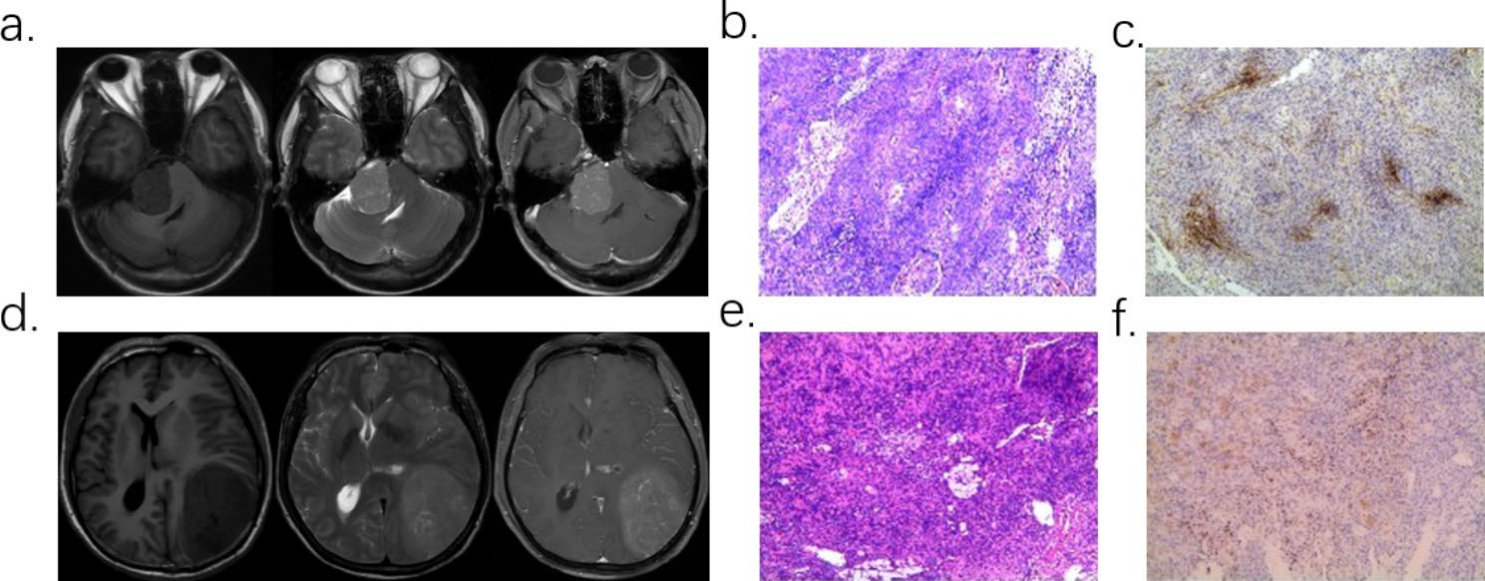
Two case examples. **a-c**. Patient with WHO I meningioma, Ki-67 was 5-10%. **a**. T1WI, T2WI, T1CE sequence MRI image. **b-c**. Pathological sections and immunohistochemical staining. **d-f**. Patient with WHO II meningioma, Ki-67 was 2-3%. **d**. T1WI, T2WI, T1CE sequence MRI image. **e-f**. Pathological sections and immunohistochemical staining

Two radiologists with over five years of experience in image reading systematically analyzed eight radiological features, including peritumoral edema, cerebrospinal fluid (CSF) space surrounding the tumor, capsular enhancement, heterogeneous enhancement, intratumoral necrosis, cross-flax or tentorium, dural tail, and surrounding invasion. Univariate and multivariate analyses were performed to identify clinical and radiological features significantly correlated with different grade, Ki-67, and their combination groups. A *P* value less than 0.05 was considered statistically significant in both univariate and multivariate analyses for clinical and radiological features. Features that showed statistical significance in both univariate and multivariate analyses were selected to build the CRR model. Figure 1 shows two case examples.

### Radiomic features extraction

The “pyradiomics” package (http://www.radiomics.io/pyradiomics.html) [12], which is based on IBSI in Python was used to extract the radiomic features. These features included shape features, first-order radiomic features, and higher-order radiomic features from five different matrices: the gray-level co-occurrence matrix (GLCM), gray-level run length matrix (GLRLM), gray-level size zone matrix (GLSZM), gray-level dependence matrix (GLDM), and neighborhood gray-tone difference matrix (NGTDM). Referring to the example YAML files provided by ‘pyradiomics’ package, which are suitable for extracting features from both 2D and 3D ROIs, extract radiomic features form both types of ROIs. The detail of two YAML files is shown in supplementary material. The radiomic features were standardized by moving the mean and scaling to the unit variance.

### Radiomic feature selection and development of radiomic models

**Fig. 2:**
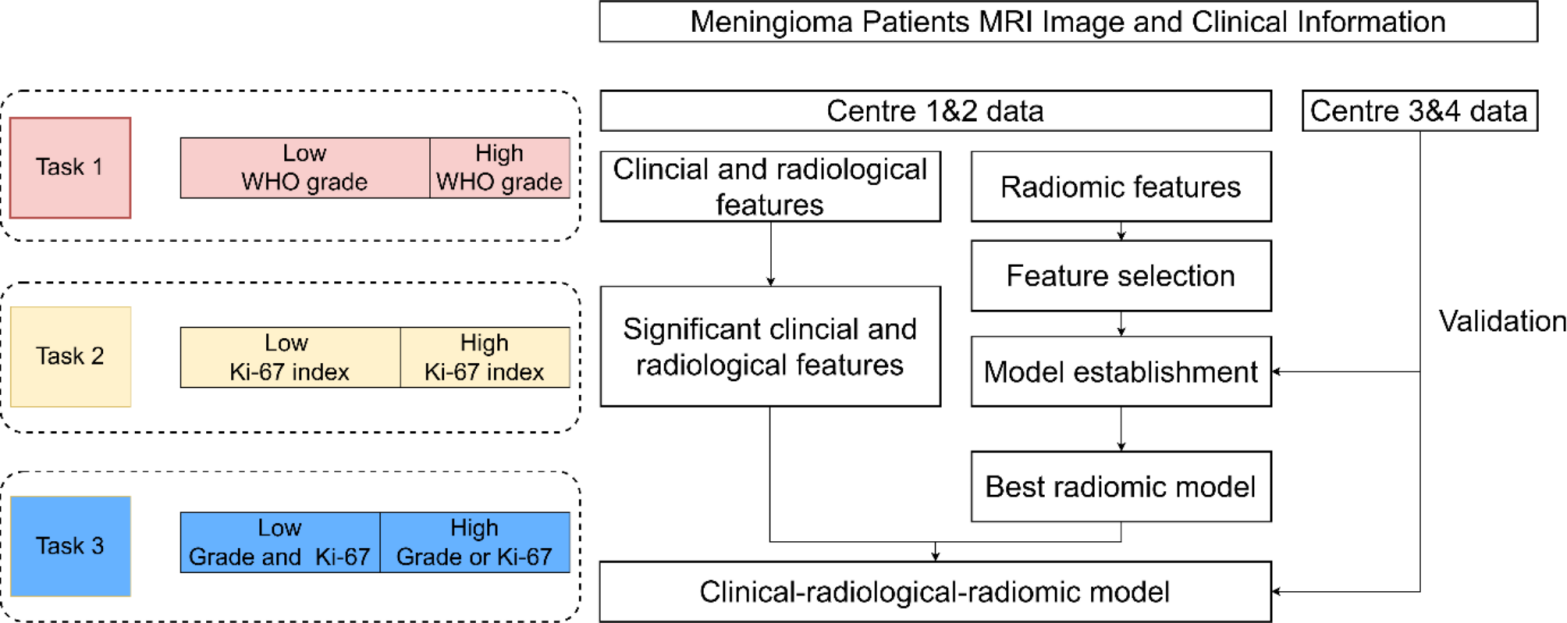
The flowchart of necessary steps in this study for each task

As mentioned above, this study develops and validates machine learning models to accomplish three tasks: predict WHO grade, Ki-67 index, and the combined of grade and Ki-67 of meningioma. Figure 2 describes the workflow of this study.

The extensive number of the radiomic features must be selected properly to avoid overfitting the machine learning algorithms. The T-test and the minimum redundancy maximum relevance (MRMR) methods were used in the training set to preliminarily select the relatively important features. Based on the number of training set patients, the top 25 features were selected.

Due to the low proportion of high grade and high Ki-67 meningioma patients, the synthetic minority oversampling technique (SMOTE) was employed to obtain smoother data for training the model after the preliminary feature selection process in training set.

The feature selection method and machine learning algorithm we used have been proved to have good results in previous studies, including least absolute shrinkage and selection operator (LASSO), mutual Information (MI), recursive feature elimination (RFE), random forest (RF), and analysis of variance (ANOVA). For RFE, MI, and RF, 20 features were selected for model establishment [13].Eight supervised machine learning algorithms were applied. These classifiers were support vector machines (SVM), logistic regression (LR), Naive Bayes (NB), decision tree (DT), random forest (RF), extreme gradient boosting (XGBOOST), light gradient boosting machine (LGBM), and k nearest neighbors (KNN). Machine learning models use basic default parameters.

In combination with three MRI sequences, two types of ROIs, five feature selection methods, and eight classifiers, 240 (3 × 2 × 5 × 8 = 240) models were built for each task. The nomenclature of each model combined four elements, including the sequence, ROI dimension, feature selection method, and machine learning algorithm. For example, T1CE-2D-LASSO-SVM was a model trained by a support vector machines classifier with features selected by the LASSO, which feature an extract from the largest section of the T1CE sequence.

5-fold cross-validation repeated 5 times was carried out on the training set. Models’ predictive performance and stability were evaluated by cross-validation area under the curve (AUC), and the cross-validation AUCs relative standard deviation (RSD) of the cross-validation AUCs, respectively. RSD was defined as RSD= (Standard Deviation of AUC / Mean AUC) ×100%. Models with an RSD value less than 0.1 were considered stable. After comparing the cross-validation AUC and RSD, the best radiomic model for each task was selected.

The performance of the selected radiomic model in the validation set was assessed using AUC, sensitivity, and specificity.

### Development clinical-radiological-radiomic models

The clinical-radiological-radiomic (CRR) models employed the classifier of the best radiomic models and were trained by combining significant clinical-radiological features with the radiomic features of the best radiomic model. The performances of the CRR models were evaluated with AUC, sensitivity, and specificity in the validation set. Additionally, Delong test was used to compare the performance of the CRR models with the radiomic models in the validation set.

### Statistical analysis

Image preprocessing, radiomic features extraction, features selection, model establishment, and model validation were performed using Python (version 3.9). Image preprocessing and radiomic feature extraction were performed using the “simpleITK” and “pyradiomics” package. The “sklearn” package played a central role in feature selection and model establishment. We employed the “pymrmr” package for MRMR feature selection. For LGBM modeling, we utilized the “lightgbm” package. Details of the used packages and module information can be found in the supplementary materials.

Categorical variables were presented with percentages and frequencies, whereas continuous variables were denoted as mean ± standard deviation or median (interquartile range, IQR) according to whether they conformed to the normal distribution. In addition, the Mann-Whitney U test and the Chi-squared test were used to assess the differences between the training and the test sets for continuous and categorical variables, respectively. In the univariate and multivariate analysis, the chi-square test and logistic regression were employed to assess the associations between classification and clinical and imaging features, while a p-value less than 0.05 were considered statistically significant. The statistical analysis was performed with the IBM SPSS v 24.0 (IBM Corp, Armonk, New York) software package.

## Results

### Patient characteristics

Patient baseline clinical characteristics and demographics were summarized in Table 1. Of all the patients, 92 (29.4%) were male. Furthermore, the patients had a median age (inter-quartile range [IQR]) of 58.00 (50.00–66.00) years. A total of 224 patients (median [IQR]: 56.0 [48.3–64.0] years, 64 [28.6%] males) were included in the training set, while 89 patients (median [IQR]: 62.0 [53.0-70.5] years, 28 [31.5%] males) were included in the replication set.

The vast majority of the included tumors were histologically confirmed as low-grade meningioma (77.6%). The Ki-67 index of tumor specimens ranged from 1% to 25% with a median (inter-quartile range [IQR]) index of 2% (1–5%). Among all the patients, 216 (69.0%) had a low Ki-67 index, while 97 patients (31.0%) had a high Ki-67 index.

**Table 1:** Clinical characteristics of the study population in discovery and validation set

	Training set (n = 224)	Validation set (n = 89)
Age, years	56.0 [48.3–64.0]	62.0 [53.0-70.5]
Sex, n		
Female	160 (71.4%)	61 (68.5%)
Male	64 (28.6%)	28 (31.5%)
WHO grade, n		
I	181 (80.8%)	62 (69.7%)
II/ III	43 (19.2%)	27 (30.3%)
Ki-67 index, n		
Ki-67 < 5	157 (70.1%)	59 (66.3%)
Ki-67 ≥ 5	67 (29.9%)	30 (33.7%)

### Clinical and radiological findings

From the univariate and multivariate results, it can be inferred that the presence of heterogeneous enhancement was significantly associated with a high grade and high Ki-67 index. Surrounding invasion was also significantly associated with high grade, while age, peritumoral edema, CSF space surrounding tumor associated with high Ki-67 index. Age and gender are associated with the malignant biological behavior of meningiomas when considering both grade and Ki-67 indicators comprehensively. The results of the univariate and multivariate analyses are demonstrated in Supplementary Table S1-2.

### Radiomic models establishment and selection

A total of 1218 and 1781 radiomic features were initially retrieved from 2D ROI and 3D ROI. Excellent consistency was found in radiomic features in interobserver agreement between the two radiologists. For T1CE-2D, T1CE-3D, T2WI-2D, T2WI-3D, T1WI-2D, and T1WI-3D, the number of features with satisfactory agreement (ICC ≥ 0.8) was 780,1598, 857, 1703, 967, and 1688, respectively.

After trying several algorithm combinations (240 for each task), we compared the cross-validation AUC of each combination and selected the best radiomic models. Cross-validation AUC and RSD heat map are depicted in Supplementary Figs. 1–6.

Many models showed good prediction efficiency in predict the grade of meningioma, T1CE-2D-LASSO-LR model was considered the best model. This model achieved the highest cross-validation AUC (0.857 95% CI:0.836–0.879) in training set. In the validation set, AUC, sensitivity, and specificity were 0.829, 0.815, and 0.661, respectively.

In this study, the T1CE-3D-LASSO-NB model was identified as the most effective radiomic model for predict Ki-67 index of meningioma, with the highest training set cross-validation AUC of 0.798 (95% CI: 0.745–0.854). In the validation set, AUC, sensitivity, and specificity were 0.752, 0.700, and 0.780, respectively.

Based on the known evidence, meningioma with high Ki-67 index or high grade typically require more aggressive treatment and shorter-term imaging follow-up. The T1CE-2D-LASSO-LR model was identified as the best model for predicting the combination status of grade and Ki-67, with a cross-validation AUC of 0.888 (95% CI: 0.856–0.923). In the validation set, AUC, sensitivity, and specificity were 0.904, 0.827, and 0.604, respectively.

**Fig. 3:**
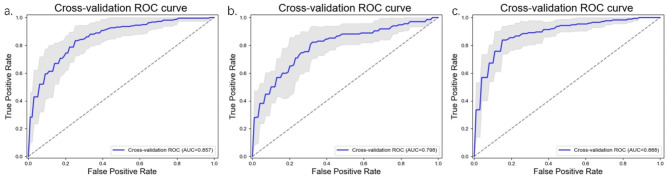
Cross-validation Receiver operating characteristic of the selected models in training set. **(a)**. T1CE-2D-LASSO-LR model predict meningioma grade. **(b)**. T1CE-3D-LASSO-NB model predict meningioma Ki-67 index. **(c)**. T1CE-2D-LASSO-LR model predict meningioma grade & Ki-67 index. 2D, two-dimensional; 3D, three-dimensional; LASSO, least absolute shrinkage and selection operator; NB, Naive Bayes; LR, logistic regression

**Fig. 4:**
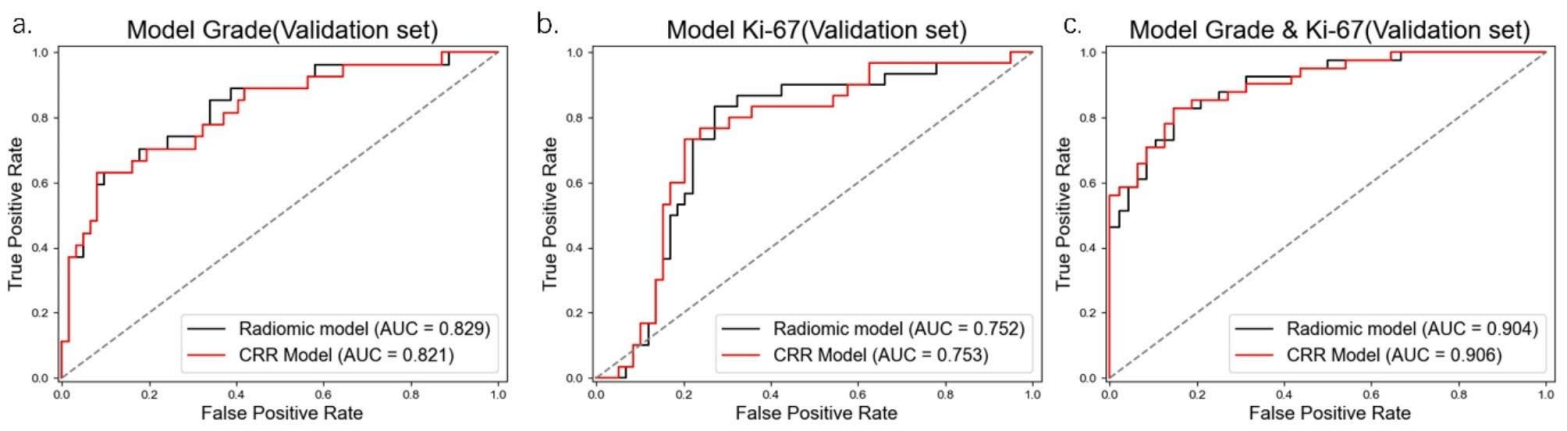
Receiver operating characteristic curves of a selected radiomic model and Clinical-radiological-radiomic models in validation set

All selected models have RSD values less than 0.1, indicating the predictive performance of these models was stable during cross-validation. Table 2 present the performance of the selected radiomic models. Figure 3 displays the cross-validation ROC curves of radiomic models in the training set. Figure 4 displays the ROC curves of the radiomic models in the validation set.

### Performances of clinical-radiological-radiomic model

CRR models only marginally improved model performance in predicting meningioma Ki-67 index and in predicting combined grade and Ki-67 index in the validation set (0.753 vs. 0.752, 0.906 vs. 0.904). For model predict grade, RCC model achieved a slightly lower AUC in the replication set (0.829 vs. 0.821). The Delong test of those CRR models with the radiomic models detected no significant differences (*P* < 0.05). Table 2 list the performance of the CRR models in the validation set. Figure 4 displays the ROC curves of the CRR models in the validation set.

**Table 2:** Selected radiomic machine learning model and clinical-radiological-radiomic machine learning model performance in training and validation set

Models (Sequence-ROI-FS-ML)		Training set (n = 224)		Validation set (n = 89)				
		CV-AUC (95%CI)	CV-RSD	AUC (95%CI)	Sensitivity (95%CI)	Specificity (95%CI)	Delong test	
							Z-score	p
Grade (T1CE-2D-LASSO-LR)	Radiomic	0.857 (0.836–0.880)	0.060	0.829 (0.786–0.863)	0.815 (0.613–0.930)	0.661 (0.529–0.774)	-1.424	0.154
	CRR			0.821 (0.759–0.858)	0.778 (0.573–0.906)	0.629 (0.497–0.746)		
Ki-67 (T1CE-3D-LASSO-NB)	Radiomic	0.798 (0.745–0.854)	0.090	0.752 (0.693–0.776)	0.700 (0.504–0.846)	0.780 (0.649–0.873)	0.073	0.942
	CRR			0.753 (0.692–0.782)	0.733 (0.538–0.870)	0.763 (0.631–0.860)		
Grade & Ki-67 (T1CE-2D-LASSO-LR)	Radiomic	0.888 (0.856–0.923)	0.051	0.904 (0.876–0.914)	0.927 (0.790–0.981)	0.604 (0.453–0.739)	0.233	0.816
	CRR			0.906 (0.876–0.916)	0.878 (0.730–0.954)	0.708 (0.557–0.826)		

ROI, regions of Interest; FS, feature selection; ML, machine learning algorithm; 2D, two-dimensional; 3D, three-dimensional; LASSO, least absolute shrinkage and selection operator; AUC, area under the curve; RSD, relative standard deviation; NB, Naive Bayes; LR, logistic regression

## Discussion

In this study, we constructed and selected radiomic models to predict meningioma grade, Ki-67 index, and combination of the two indices. The relationship between clinical-radiological features and meningioma grade or Ki-67 index was explored, and by combining meaningful features, the clinical-radiological-radiomic models were established.

Some previous studies have used radiomic based machine learning models to predict the meningioma grade [14]. Chen [15] et al. applied linear discriminant analysis (LDA) and a support vector machine (SVM) to construct a radiomic model to predict of the grade of meningioma. It was found that the best model was LASSO + LDA, with an accuracy of 75.6%. Duan [16] et al. collected a more balanced number of patients with high and low grade meningiomas, yielding more plausible results. Han [17] et al. compared different classifiers used to train the models, found that SVM model performed better. Various radiomic based machine learning models were also built to predict the Ki-67 index of meningiomas. Khanna [10] et al. extracted features from multiple MR sequences, and the AUC of the test set for predicted high Ki-67 meningiomas in WHO I grade meningiomas was 0.84. Zhao [11] et al. predicted the Ki-67 index for all meningiomas obtained AUC of 0.837 and 0.700 on the internal and external validation sets. These studies suggest that machine learning models based on radiomic features can contribute to predict the grade and Ki-67 index of meningioma. Due to space limitations, not all studies have been listed, the summarizes of the relevant studies listed on Supplementary Table S3.

From the proposed work, it can be concluded that the radiomic features extracted from T1CE sequences were more effective than those from other sequences in establishing models to predict the meningioma grade, Ki-67 index and combination of the two indices. The underlying reason for this effect is likely that the T1CE sequence contains more information [15, 18]. Some studies have improved prediction performance by input features from multiple sequence, while our study only evaluates the radiomic features from single sequence. This is because different central scanning protocols make the model of a single sequence more generalizable, and when multiple sequences are input together, the radiomic features extracted from different sequences of the same patient may have high collinearity, excessive number of features and relatively small number of individuals may lead to difficulty in feature selection or loss of important information. In terms of the work flow of segmentation, previous studies have employed methods such as single researchers, double researchers, or expert assessment following double researchers. In our study, two researchers segmented the ROIs of all patient. and the ICC of the extracted radiomics features was calculated based on the segmentation results of the two researches in the training set. We selected features with an ICC greater than 0.80 for further analysis, aiming to enhance the reproducibility of radiomics features selected for modeling. Both 2DRO and 3DROI performed well in this study, some research suggests that radiomic models based on 2D ROI were better than that based on 3D ROI [16, 18, 19]. The feature selection methods and machine learning algorithms used in our research have yielded good results in previous studies and are commonly used. LASSO feature selection methods were also found to be more effective than other feature selection methods in this study, while LR and NB machine learning algorithms obtained better results than other machine learning algorithms. These findings were consistent with the results of some previous studies [15, 20] and highlight the importance of evaluating different feature selection approaches and machine learning algorithms to determine which is most suitable to the specific data being analyzed. In previous studies, radiomics machine learning models used to predict meningioma grade or Ki-67 index have been observed to show significant differences in AUC between the training and validation sets [11, 15], the potential reason could be overfitting caused by the lower incidence rate of high grade or Ki-67 meningioma and relatively small sample size of the study. In our study, we only found the grade model validation set AUC is slightly below the 95% CI of the CV-AUC, however there was partial overlap between the training and validation 95% CI, thus, the results of the model predicting grade are considered acceptable.

In this study, the relationship between the biological behavior of meningioma and clinical-radiological features was systematically analyzed, and eight features that reported to be correlated with meningioma grade and Ki-67 index [21–27] were included. Univariate and multifactor analysis revealed that meningioma with high grade or high Ki-67 index were more likely to present heterogeneous enhancement, and those with high grade were more likely to present surrounding invasion, while patients with Ki-67 index were more likely to present peritumoral edema and CSF space surrounding tumor. These radiological feature findings are consistent with previously reported studies [27, 28]. In many radiomics studies, clinical and radiological features have been widely used to improve the performance of the predicting models [11, 16]. However, in this study, statistically significant radiological features did not significantly improve the predictive performance of models. These clinical and radiological features can be double-edged swords, on the one hand, unlike higher-order radiomic features, the relationship between these features and tumor biological behavior is well established, providing a clearer interpretation, on the other hand, the introduction of these features may add subjective information to an objective quantitative model. Furthermore, some radiomic features are derived from the quantification of radiological features.

Compared to similar studies, this study had some improvements. Firstly, this study trains and validates on multicenter data with the aim of improving the model’s generalization and increasing its clinical utility. Secondly, as there are currently no uniform guidelines for the feature selection and machine learning algorithm in the radiomic workflow [29], with reference to Zhang’s study [30], different combinations of different sequences, ROIs, feature selection methods, and machine learning algorithms were used to build models. Thirdly, the SMOTE technique was used in the training set to obtain more balanced data for the training of the models [8], and cross-validation on training set was used to reduce the effect of different groupings in selecting suitable models, Those models selected by comparing the cross-validation AUC and RSD value rather than empirically selecting commonly used modeling methods are not always suitable for particular data. Finally, this study simultaneously predicted the grade and Ki-67 index, obtaining more information from a single MRI examination. Furthermore, this study has developed more clinically justifiable models from the perspective of combining both the grade and Ki-67 index.

However, the study had some limitations. Firstly, the retrospective nature of the study and the inclusion of only patients who underwent meningioma resection may have introduced selection bias. Secondly, the study did not adequately explore the relationship between radiological features and radiomics features, which may have limited the interpretation of the findings. Finally, this study only used a single MRI sequence for modeling, the combination of sequences needs to be evaluated. Future studies could incorporate more sequences and experiments with semi-automatic segmentation of ROIs and employ deep learning methods.

## Conclusion

In conclusion, MRI radiomic machine learning models could effectively predict the biological behavior of meningioma from the perspective of grade, Ki-67 index and the combination of both. The latter model could be valuable for improving the predictive sensitivity and comprehensiveness of predicting the malignant biological behavior of meningioma.

### Electronic supplementary material

Below is the link to the electronic supplementary material.

Supplementary Material 1

## Data Availability

The data sets generated and analyzed during the current study are available from the corresponding author on reasonable request.

## References

[1] Louis DN, Perry A, Wesseling P, Brat DJ, Cree IA, Figarella-Branger D (2021). The 2021 WHO classification of tumors of the Central Nervous System: a summary. Neurooncology.

[2] Goldbrunner R, Stavrinou P, Jenkinson MD, Sahm F, Mawrin C, Weber DC (2021). EANO guideline on the diagnosis and management of meningiomas. Neurooncology.

[3] Ogasawara C, Philbrick BD, Adamson DC, Meningioma. A review of Epidemiology, Pathology, diagnosis, treatment, and future directions. Biomedicines. 2021;9(3). 10.3390/biomedicines9030319 PMC8004084 33801089

[4] Menon SS, Guruvayoorappan C, Sakthivel KM, Rasmi RR (2019). Ki-67 protein as a tumour proliferation marker. Clin Chim Acta.

[5] Nowak-Choi K, Palmer JD, Casey J, Chitale A, Kalchman I, Buss E (2021). Resected WHO grade I meningioma and predictors of local control. J Neurooncol.

[6] Liu N, Song SY, Jiang JB, Wang TJ, Yan CX (2020). The prognostic role of Ki-67/MIB-1 in meningioma: a systematic review with meta-analysis. Medicine.

[7] Apra C, Peyre M, Kalamarides M (2018). Current treatment options for meningioma. Expert Rev Neurother.

[8] Hu J, Zhao Y, Li M, Liu J, Wang F, Weng Q (2020). Machine learning-based radiomics analysis in predicting the meningioma grade using multiparametric MRI. Eur J Radiol.

[9] Neromyliotis E, Kalamatianos T, Paschalis A, Komaitis S, Fountas KN, Kapsalaki EZ et al. Machine learning in Meningioma MRI: past to Present. Narrative Rev J Magn Reson Imaging: JMRI. 2020. 10.1002/jmri.27378 33006425

[10] Khanna O, Fathi Kazerooni A, Farrell CJ, Baldassari MP, Alexander TD, Karsy M (2021). Machine learning using Multiparametric magnetic resonance imaging Radiomic feature analysis to Predict Ki-67 in World Health Organization Grade I Meningiomas. Neurosurgery.

[11] Zhao Y, Xu J, Chen B, Cao L, Chen C. Efficient prediction of Ki-67 Proliferation Index in Meningiomas on MRI: from traditional radiological findings to a machine learning Approach. Cancers. 2022;14(15). 10.3390/cancers14153637 PMC9330288 35892896

[12] van Griethuysen JJM, Fedorov A, Parmar C, Hosny A, Aucoin N, Narayan V (2017). Computational Radiomics System to Decode the Radiographic phenotype. Cancer Res.

[13] Joo L, Park JE, Park SY, Nam SJ, Kim YH, Kim JH (2021). Extensive peritumoral edema and brain-to-tumor interface MRI features enable prediction of brain invasion in meningioma: development and validation. Neurooncology.

[14] Neromyliotis E, Kalamatianos T, Paschalis A, Komaitis S, Fountas KN, Kapsalaki EZ (2022). Machine learning in Meningioma MRI: past to Present. A narrative review. J Magn Reson Imaging: JMRI.

[15] Chen C, Guo X, Wang J, Guo W, Ma X, Xu J (2019). The diagnostic value of Radiomics-Based machine learning in Predicting the Grade of Meningiomas using Conventional magnetic resonance imaging: a preliminary study. Front Oncol.

[16] Duan C, Zhou X, Wang J, Li N, Liu F, Gao S (2022). A radiomics nomogram for predicting the meningioma grade based on enhanced T(1)WI images. Br J Radiol.

[17] Han Y, Wang T, Wu P, Zhang H, Chen H, Yang C (2021). Meningiomas: preoperative predictive histopathological grading based on radiomics of MRI. Magn Reson Imaging.

[18] Li X, Lu Y, Xiong J, Wang D, She D, Kuai X (2019). Presurgical differentiation between malignant haemangiopericytoma and angiomatous meningioma by a radiomics approach based on texture analysis. J Neuroradiology = Journal de Neuroradiologie.

[19] Gu H, Zhang X, di Russo P, Zhao X, Xu T. The Current State of Radiomics for Meningiomas: Promises and Challenges. 2020;10. 10.3389/fonc.2020.567736 PMC7653049 33194649

[20] Lu Y, Li B, Huang H, Leng Q, Wang Q, Zhong R (2022). Biparametric MRI-based radiomics classifiers for the detection of prostate cancer in patients with PSA serum levels of 4∼10 ng/mL. Front Oncol.

[21] Kim BW, Kim MS, Kim SW, Chang CH, Kim OL (2011). Peritumoral brain edema in meningiomas: correlation of radiologic and pathologic features. J Korean Neurosurg Soc.

[22] Sun SQ, Kim AH, Cai C, Murphy RK, DeWees T, Sylvester P (2014). Management of atypical cranial meningiomas, part 1: predictors of recurrence and the role of adjuvant radiation after gross total resection. Neurosurgery.

[23] Barresi V, Lionti S, Caliri S, Caffo M (2018). Histopathological features to define atypical meningioma: what does really matter for prognosis?. Brain Tumor Pathol.

[24] Smirniotopoulos JG, Murphy FM, Rushing EJ, Rees JH, Schroeder JW (2007). Patterns of contrast enhancement in the brain and meninges. Radiographics: A Review Publication of the Radiological Society of North America Inc.

[25] Phuttharak W, Boonrod A, Thammaroj J, Kitkhuandee A, Waraasawapati S (2018). Preoperative MRI evaluation of meningioma consistency: a focus on detailed architectures. Clin Neurol Neurosurg.

[26] Deguchi S, Nakashima K, Nakasu Y, Mitsuya K, Hayashi N, Ito I (2020). A practical predictor of the growth potential of benign meningiomas: hypointensity of surface layer in T2-weighted magnetic resonance imaging. Clin Imaging.

[27] Nakamura M, Roser F, Michel J, Jacobs C, Samii M (2003). The natural history of incidental meningiomas. Neurosurgery.

[28] Harter PN, Braun Y, Plate KHJCCO. Classification of meningiomas—advances and controversies. 2017. 2017:3. 10.21037/cco.2017.05.02 28595423

[29] Lambin P, Leijenaar RTH, Deist TM, Peerlings J, de Jong EEC, van Timmeren J (2017). Radiomics: the bridge between medical imaging and personalized medicine. Nat Reviews Clin Oncol.

[30] Zhang Y, Zhang B, Liang F, Liang S, Zhang Y, Yan P (2019). Radiomics features on non-contrast-enhanced CT scan can precisely classify AVM-related hematomas from other spontaneous intraparenchymal hematoma types. Eur Radiol.

