# Supplementary material for: MRI-based machine learning models predict the malignant biological behavior of meningioma
Source: BMC Med Imaging. 2023 Sep 27;23:141. doi: 10.1186/s12880-023-01101-7 (PMC10537075; doi:10.1186/s12880-023-01101-7)
Supplement: Supplementary file 1 — Supplementary Material 1 [file 12880_2023_1101_MOESM1_ESM.docx]

Supplementary Table S1. Univariate statistical analyses of clinical and radiological features.

|  | Grade | | | Ki-67 | | | Grade& Ki-67 | | |
| --- | --- | --- | --- | --- | --- | --- | --- | --- | --- |
|  | II/III  (n=43) | I  (n=181) | *p* | Ki-67≥5%  (n=67) | Ki-67<5%  (n=157) | *p* | One high  (n=87) | Both low  (n=137) | *p* |
| Age | 52.0[45.0-65.0] | 58.0[50.0-64.0] | 0.135 | 52.0[45.0-62.0] | 58.0[50.0-65.0] | *0.042* | 52.0 [45.0-63.0] | 58.0[50.0-65.0] | *0.013* |
| Female | 27 (62.8%) | 133 (73.5%) | 0.189 | 41 (61.2%) | 119 (75.8%) | *0.035* | 51 (58.6%) | 109 (79.6%) | *<0.001* |
| Heterogeneous enhancement | 39 (92.7%) | 101 (55.8%) | *<0.001* | 59 (88.1%) | 81(51.6%) | *<0.001* | 78 (89.7%) | 62 (45.3%) | *<0.001* |
| Peritumoral edema | 22 (51.2%) | 70 (38.7%) | 0.168 | 43 (64.2%) | 49 (31.2%) | *<0.001* | 51 (58.6%) | 41 (29.9%) | *<0.001* |
| Intratumoral Necrosis | 16 (37.2%) | 32 (17.7%) | *0.007* | 24 (35.8%) | 24 (15.3%) | *<0.001* | 30 (34.5%) | 18 (13.1%) | *<0.001* |
| Capsular enhancement | 7 (16.3%) | 56 (30.9%) | 0.060 | 23 (34.3%) | 40 (25.5%) | 0.196 | 24 (27.6%) | 39 (28.5%) | 1.000 |
| CSF space surrounding tumor | 16 (39.2%) | 71 (39.2%) | 0.863 | 19(28.4%) | 68 (38.8%) | *0.037* | 29 (33.3%) | 58 (42.3%) | 0.206 |
| Dural tail | 16 (37.2%) | 40 (23.2%) | 0.080 | 24 (35.8%) | 32 (21.7%) | *0.031* | 32 (36.8%) | 26 (19.0%) | *0.005* |
| Cross flax or entorium | 13 (30.2%) | 54 (29.8%) | 1.000 | 20 (29.9%) | 47 (29.9%) | 1.000 | 25 (28.7%) | 42 (30.7%) | 0.881 |
| Surrounding invasion | 15 (34.1%) | 34 (18.3%) | *0.038* | 18 (26.9%) | 31 (19.7%) | *0.289* | 24 (27.6%) | 25 (18.2%) | 0.135 |

Supplementary Table S2. Multivariate statistical analyses of clinical and radiological features.

|  | Variables  (High Grade vs. Low Grade) | | | Variables  (High Ki-67 vs. Low Ki-67) | | Variables  (one high vs. Both low) | |
| --- | --- | --- | --- | --- | --- | --- | --- |
|  | OR,95CI | *p* | OR | | *p* | OR | *p* |
| Age | 0.980 (0.951-1.009) | 0.168 | 0.971 (0.944-0.988) | | *0.036* | 0.964 (0.937-0.992) | *0.011* |
| Female | 0.713 (0.329-1.544) | 0.391 | 0.522 (0.252-1.082) | | 0.081 | 0.350 (0.168-0.730) | *0.005* |
| Heterogeneous enhancement | 6.488 (2.083-20.212) | *<0.001* | 5.295 (2.152-13.026) | | *<0.001* | 8.213 (3.491-19.319) | *<0.001* |
| Peritumoral edema | 0.904 (0.408-2.003) | 0.803 | 3.034 (1.449-6.355) | | *0.003* | 2.004 (0.976-4.117) | 0.058 |
| Intratumoral Necrosis | 1.678 (0.703-4.004) | 0.243 | 1.238 (0.529-2.896) | | 0.622 | 1.569 (0.659-3.736) | 0.309 |
| Capsular enhancement | 0.383 (0.149-0.986) | *0.047* | 2.371 (1.091-5.152) | | *0.029* | 1.214 (0.566-2.608) | 0.618 |
| CSF space surrounding tumor | 0.833 (0.389-1.780) | 0.636 | 0.318 (0.151-0.669) | | *0.003* | 0.429 (0.213-0.864) | *0.018* |
| Dural tail | 1.342 (0.598-3011) | 0.475 | 1.251 (0.580-2.697) | | 0.568 | 1.679 (0.789-3.571) | 0.179 |
| Cross flax or entorium | 0.903 (0.402-2.028) | 0.804 | 0.839 (0.395-1.782) | | 0.648 | 0.713 (0.341-1.492) | 0.369 |
| Surrounding invasion | 2.463 (1.034-5.587) | *0.042* | 1.764 (0.754-4.131) | | 0.191 | 2.167 (0.935-5.022) | 0.071 |

Supplementary Table S3. Summary of previous reported radiomics study predicting meningioma grade or Ki-67.

| Research groups | years | Sample Size (High vs Low) | Modality | Segmentation | ROI | Feature Selection | models | Result |
| --- | --- | --- | --- | --- | --- | --- | --- | --- |
| Grade | | | | | | | | |
| Coroller^[1]^ et al. | 2017 | 175  (72 vs 103) | T1CE | The segmentation was done by two neurosurgeons and reviewed by another neuroradiologist. | 3D | PCA, Variance Correlation | RF | Internal validation AUC=0.78. |
| Yan^[2]^ et al. | 2017 | 131  (21 vs 110) | T1WI, T2WI, FLAIR, T1CE | One investigator performed segmentation, which was later jointly re-examined and modified by two investigators | 2D | Correlation based feature selection | LR, NB, SVM | The SVM model had highest ten-fold AUC of 0.87. |
| Park^[3]^ et al. | 2018 | 194  (40 vs 154) | TICE,  ADC, FA | One neuroradiologist segmented, another confirmed | 3D | RFE | SVM, RF | RFE-SVM-SMOTE model had highest test AUC of 0.86. |
| Lu^[4]^ et al. | 2018 | 152  (64vs 88) | T1WI,  T1CE,  T2WI,  FLAIR,  DWI, ADC | Two neuro-radiologists performed segmentation. | 2D | Fisher coefficient and classification error probability, average correlation coefficient (POE +ACC) | DT, CIT, DF | DF model had highest test accuracy of 0.80. |
| Laukamp^[5]^ et al. | 2019 | 71  (25 vs 46) | T1WI, T2WI, T1CE, FLAIR, DWI, ADC | Two radiologists performed segmentation. | 3D | RF | Multivariate logistic regression analysis | Training data AUC=0.91. |
| Hamerla^[6]^ et al. | 2019 | 138  (45 vs 93) | T1WI, T2WI, T1CE, FLAIR, ADC, | One neuroradiology performed segmentation. | 3D | Mann-Whitney U test | RF, XGBOOST, SVM, MLP | The XGBOOST model had highest ten-fold AUC of 0.97. |
| Chen^[7]^ et al. | 2019 | 150  (61 vs 89) | TIWI, T1CE | Two neurosurgeons performed segmentation. | 3D | Distance correlation,  LASSO,  GBDT | LDA, SVM | The Lasso + LDA model had highest accuracy of 0.76. |
| Zhu^[8]^ et al. | 2019 | 181  (35 vs 146) | T1CE | Two radiologists performed segmentation. | 2D | RF,  SBS | LDA | Validation AUC=0.816. |
| Morin^[9]^ et al. | 2019 | 303  (125vs 179) | T1WI, T2WI,  FLAIR, DWI,  ADC, SPGR,  T1CE | Two radiologists performed segmentation. | 3D | Supervised  false-positive  avoidance  methodology | RF | Combined demographic, radiologic, radiomic model with validation AUC of 0.78. |
| Han^[10]^ et al. | 2020 | 131  (58 vs73) | T1CE, T2WI,  T1-FLAIR | The segmentation was done by a radiologist and reviewed by a neuroradiologist. | 3D | Low variance, univariate,  LASSO | DT, SVM, LR, KNN, RF, XGBOOST | SVM model had highest AUC of 0.96 in validation set. |
| Hu^[11]^ et al. | 2020 | 316  (87 vs 229) | SWI, ADC, cMR | The segmentation was done by two neurosurgeons and disagreements were resolved by another neuroradiologist. | 3D | LASSO | RF | cMRI + ADC + SWI model had highest ten-fold AUC of 0.84. |
| Chu^[12]^ et al. | 2020 | 96  (16 vs 82) | T1WI | One radiologist performed segmentation. | 3D | LASSO | LR | Test set AUC of 0.95. |
| Duan^[13]^ et al. | 2021 | 184  (94 vs 94) | T1CE | One neuroradiologist segmented, another confirmed | 2D | LASSO | SVM, DT, CIT, RF, KNN, BPNet, NB | SVM model had highest AUC of 0.88 in validation set. |
| Ki-67 | | | | | | | | |
| Khanna^[14]^ et al. | 2021 | 306  (92 vs 214) | T1WI, T2WI, T1CE, FLAIR, DWI, ADC | One neurosurgeon performed segmentation. | 3D | LASSO | SVM | Test set AUC of 0.83. |
| Zhao^[15]^ et al. | 2022 | 371  (172 vs 199) | T1CE | The segmentation was done by two neuro-radiologists  Checked by another neuro-radiologist. | 3D | LASSO, ETC, SVC | LDA | LASSO+LDA model had highest external test AUC of 0.70. |

PCA, principal component analysis; RF, random forest; AUC, area under the curve; LR, logistic regression; NB, naive Bayes; SVM, support vector machine; RFE, recursive feature elimination, LASSO, least absolute shrinkage and selection operator; SBS, sequential backward selection; LDA, linear discriminant analysis; XGBOOST, eXtreme gradient boosting; MLP, multilayer perceptron; GBDT, gradient boosting decision tree; ETC, Extra tree classifier; SMOTE, Synthetic Minority Over-sampling Technique; DT, decision tree; CIT, conditional inference tree; DF, decision forest; KNN, K-Nearest neighbors; T1WI, T1-weighted imaging; T2WI, T2-weighted imaging; T1CE, T1-weighted contrast enhanced; FLAIR, fluid light attenuation inversion recovery; DWI, diffusion-weighted imaging; ADC, apparent diffusion coefficient; SWI, susceptibility weighted imaging; cMRI, conventional magnetic resonance imaging.

[1] COROLLER T P, BI W L, HUYNH E, et al. Radiographic prediction of meningioma grade by semantic and radiomic features [J]. PloS one, 2017, 12(11): e0187908.

[2] YAN P F, YAN L, HU T T, et al. The Potential Value of Preoperative MRI Texture and Shape Analysis in Grading Meningiomas: A Preliminary Investigation [J]. Translational oncology, 2017, 10(4): 570-7.

[3] PARK Y W, OH J, YOU S C, et al. Radiomics and machine learning may accurately predict the grade and histological subtype in meningiomas using conventional and diffusion tensor imaging [J]. European radiology, 2019, 29(8): 4068-76.

[4] LU Y, LIU L, LUAN S, et al. The diagnostic value of texture analysis in predicting WHO grades of meningiomas based on ADC maps: an attempt using decision tree and decision forest [J]. European radiology, 2019, 29(3): 1318-28.

[5] LAUKAMP K R, SHAKIRIN G, BAEßLER B, et al. Accuracy of Radiomics-Based Feature Analysis on Multiparametric Magnetic Resonance Images for Noninvasive Meningioma Grading [J]. World neurosurgery, 2019, 132(366-90.

[6] HAMERLA G, MEYER H J, SCHOB S, et al. Comparison of machine learning classifiers for differentiation of grade 1 from higher gradings in meningioma: A multicenter radiomics study [J]. Magnetic resonance imaging, 2019, 63(244-9.

[7] CHEN C, GUO X, WANG J, et al. The Diagnostic Value of Radiomics-Based Machine Learning in Predicting the Grade of Meningiomas Using Conventional Magnetic Resonance Imaging: A Preliminary Study [J]. Frontiers in oncology, 2019, 9(1338.

[8] ZHU Y, MAN C, GONG L, et al. A deep learning radiomics model for preoperative grading in meningioma [J]. European journal of radiology, 2019, 116(128-34.

[9] MORIN O, CHEN W C, NASSIRI F, et al. Integrated models incorporating radiologic and radiomic features predict meningioma grade, local failure, and overall survival [J]. Neuro-oncology advances, 2019, 1(1): vdz011.

[10] HAN Y, WANG T, WU P, et al. Meningiomas: Preoperative predictive histopathological grading based on radiomics of MRI [J]. Magnetic resonance imaging, 2021, 77(36-43.

[11] HU J, ZHAO Y, LI M, et al. Machine learning-based radiomics analysis in predicting the meningioma grade using multiparametric MRI [J]. European journal of radiology, 2020, 131(109251.

[12] CHU H, LIN X, HE J, et al. Value of MRI Radiomics Based on Enhanced T1WI Images in Prediction of Meningiomas Grade [J]. Academic radiology, 2021, 28(5): 687-93.

[13] DUAN C F, LI N, LI Y, et al. Comparison of different radiomic models based on enhanced T1-weighted images to predict the meningioma grade [J]. Clinical radiology, 2022, 77(4): e302-e7.

[14] KHANNA O, FATHI KAZEROONI A, FARRELL C J, et al. Machine Learning Using Multiparametric Magnetic Resonance Imaging Radiomic Feature Analysis to Predict Ki-67 in World Health Organization Grade I Meningiomas [J]. Neurosurgery, 2021, 89(5): 928-36.

[15] ZHAO Y, XU J, CHEN B, et al. Efficient Prediction of Ki-67 Proliferation Index in Meningiomas on MRI: From Traditional Radiological Findings to a Machine Learning Approach [J]. Cancers, 2022, 14(15):


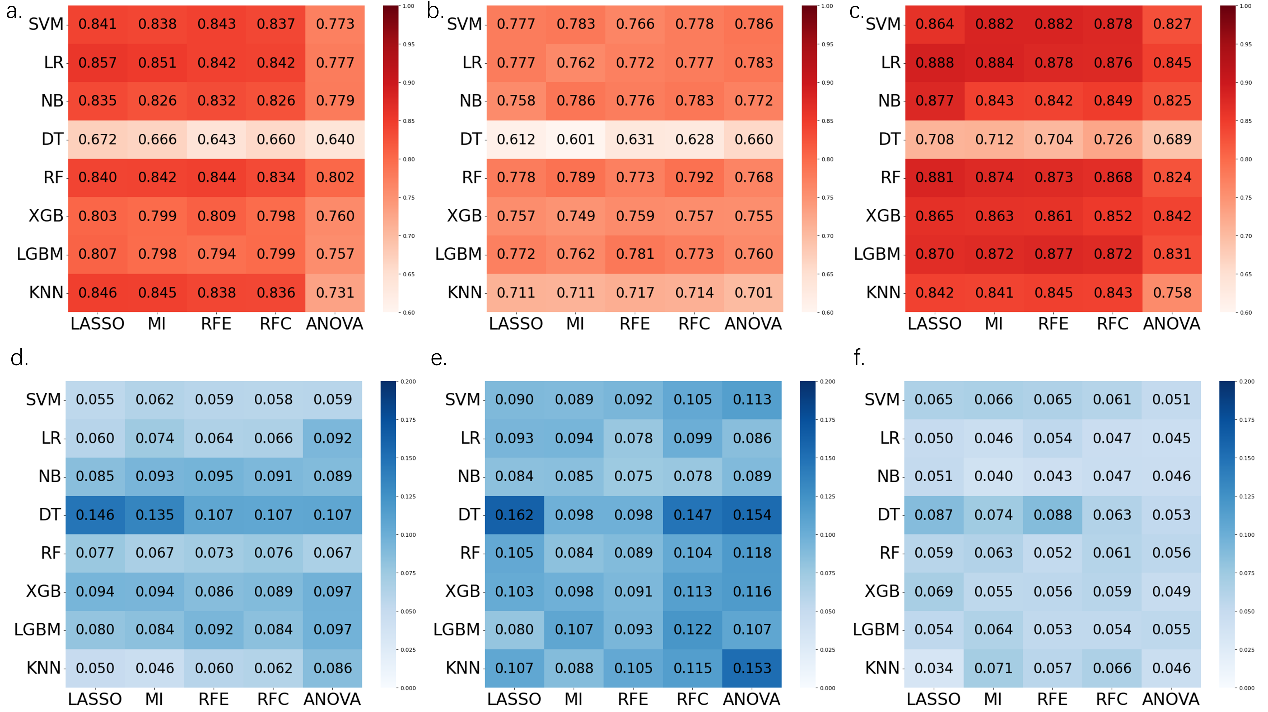


Supplementary Fig.1 Heatmaps illustrating the predictive performance of different combinations of feature selection methods (rows) and classification algorithms (columns). (a-c) Cross-validated AUC values of models predict grade, Ki-67, and combined grade & Ki-67 based on T1CE-2D on the training set. (d-f) Cross-validated RSD values of models predict grade, Ki-67, and combined grade & Ki-67 based on T1CE-2D on the training set.


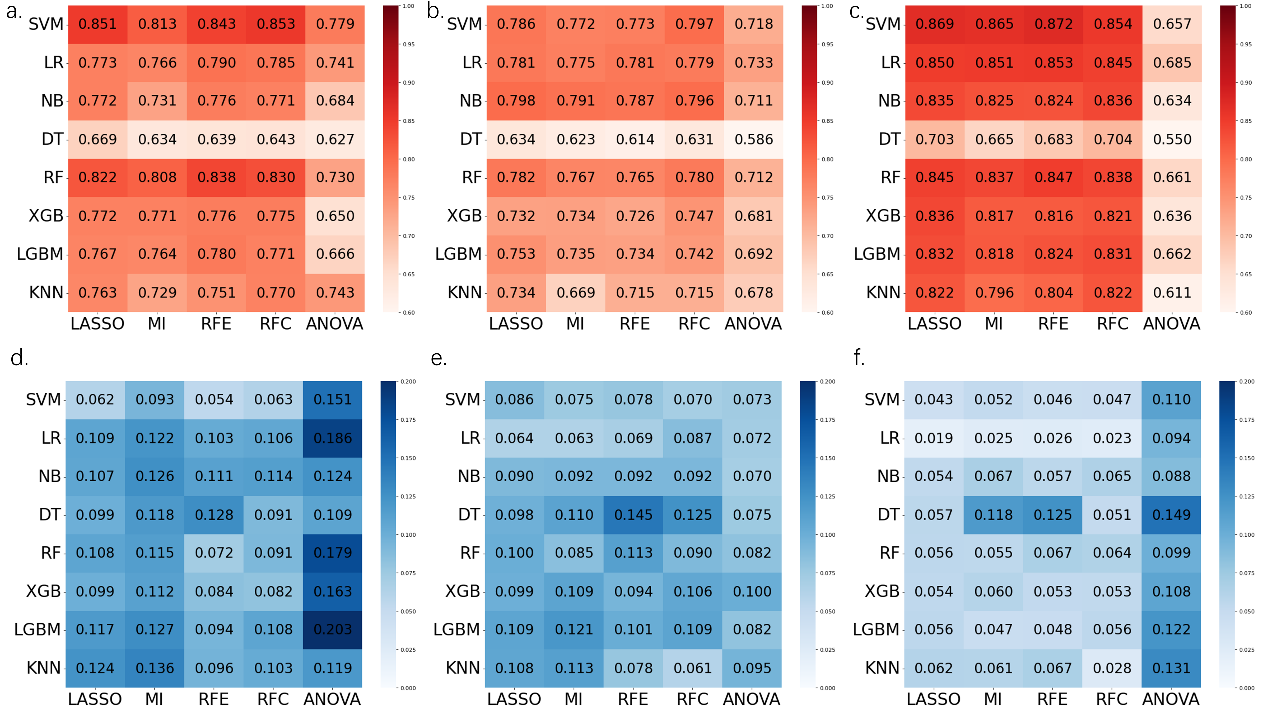


Supplementary Figure 2. Heatmaps illustrating the predictive performance of different combinations of feature selection methods (rows) and classification algorithms (columns). (a-c) Cross-validated AUC values of models predict grade, Ki-67, and combined grade & Ki-67 based on T1CE-3D on the training set. (d-f) Cross-validated RSD values of models predict grade, Ki-67, and combined grade & Ki-67 based on T1CE-3D on the training set.


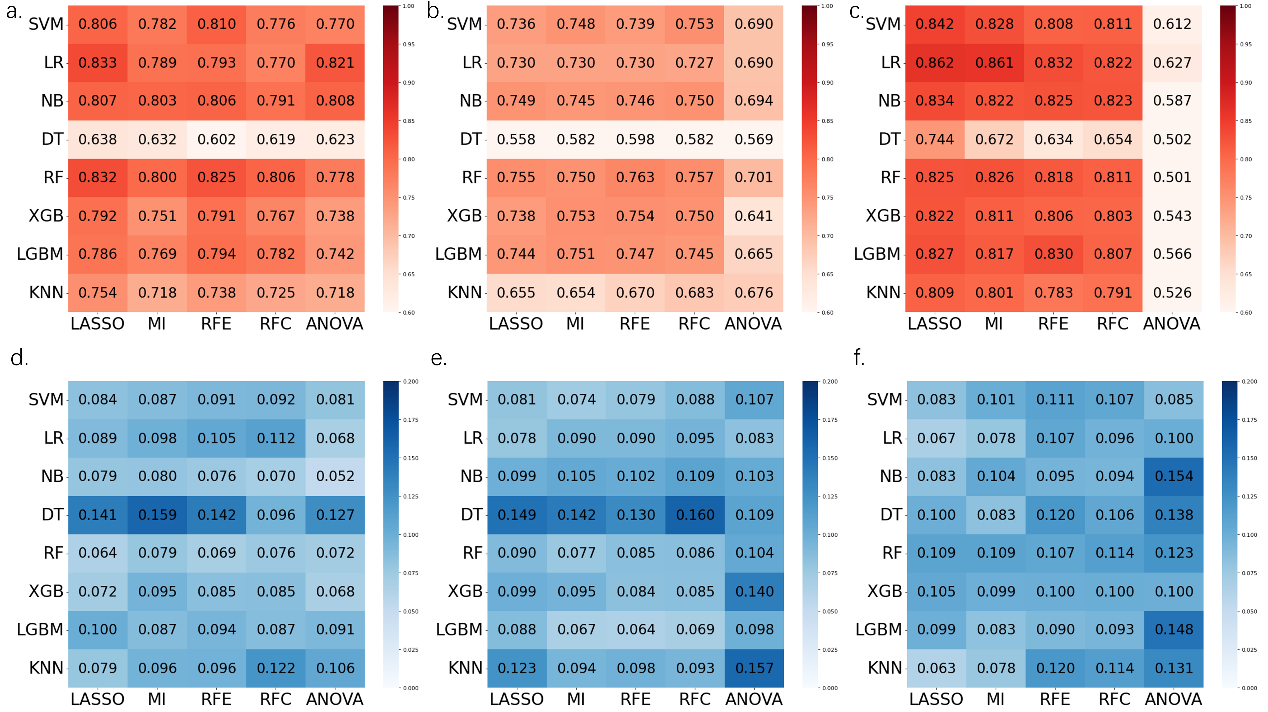


Supplementary Figure 3. Heatmaps illustrating the predictive performance of different combinations of feature selection methods (rows) and classification algorithms (columns). (a-c) Cross-validated AUC values of models predict grade, Ki-67, and combined grade & Ki-67 based on T1-2D on the training set. (d-f) Cross-validated RSD values of models predict grade, Ki-67, and combined grade & Ki-67 based on T1-2D on the training set.


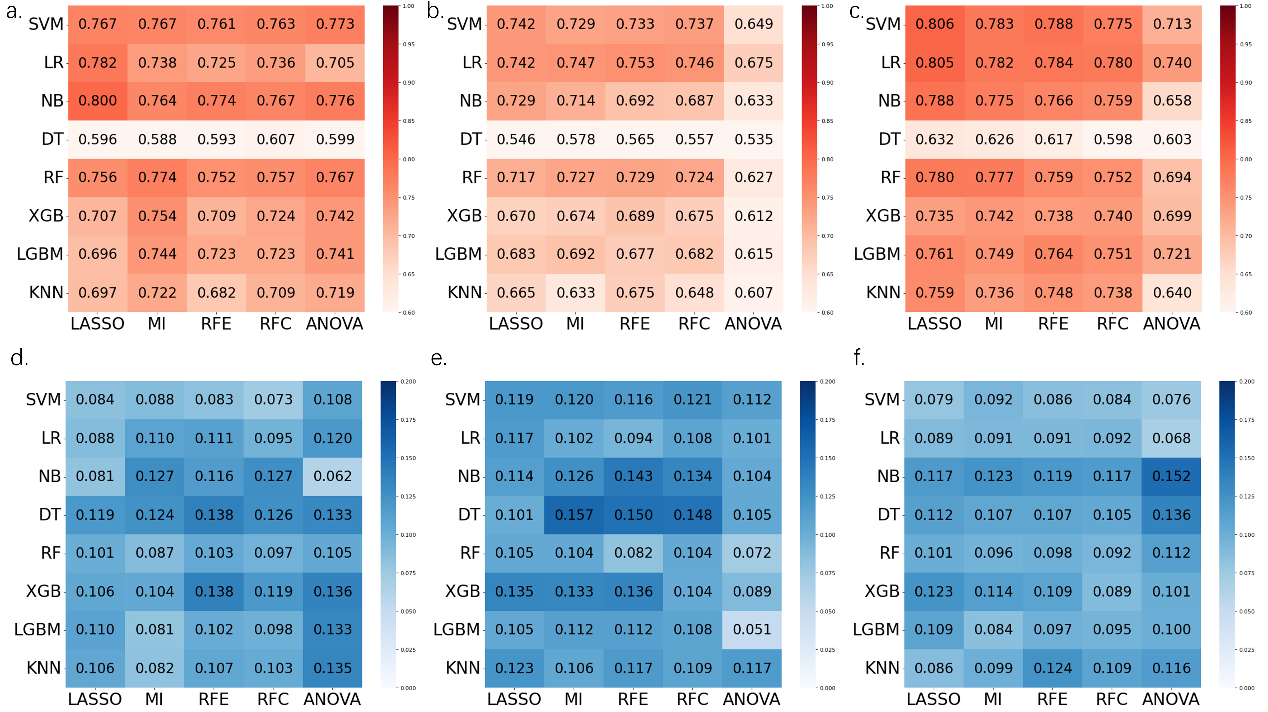


Supplementary Figure 4. Heatmaps illustrating the predictive performance of different combinations of feature selection methods (rows) and classification algorithms (columns). (a-c) Cross-validated AUC values of models predict grade, Ki-67, and combined grade & Ki-67 based on T1-3D on the training set. (d-f) Cross-validated RSD values of models predict grade, Ki-67, and combined grade & Ki-67 based on T1-3D on the training set.


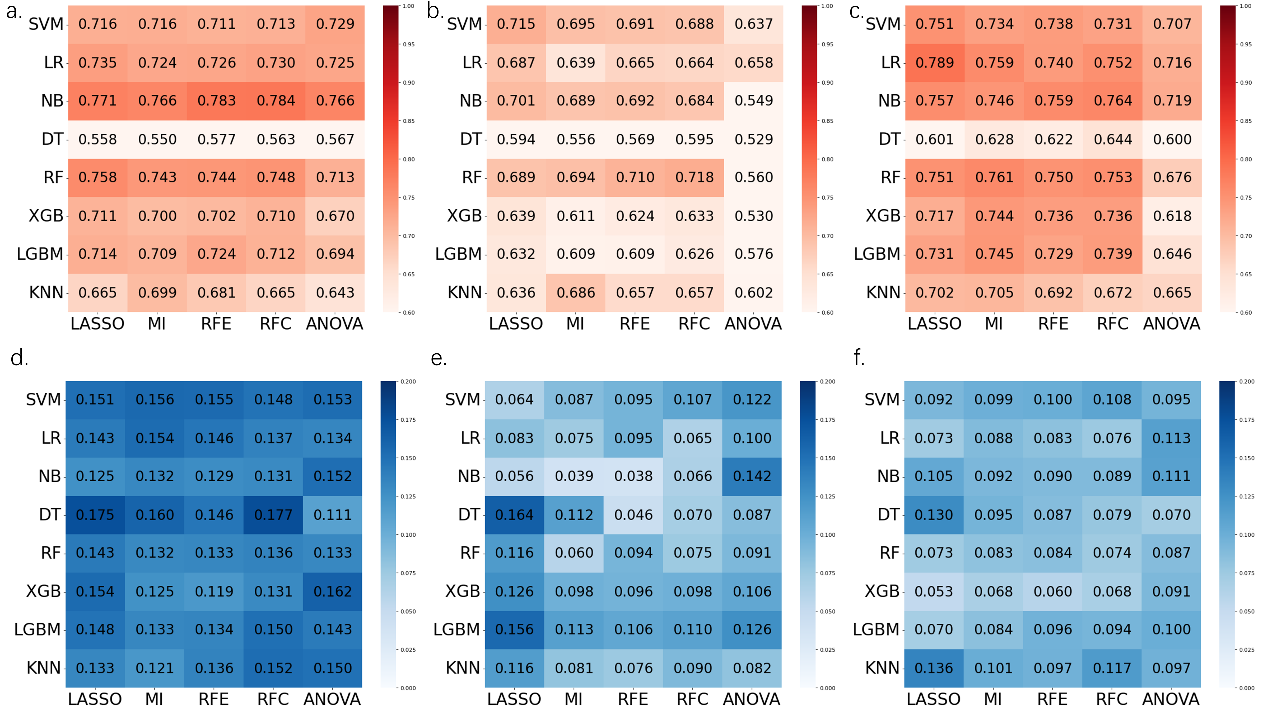


Supplementary Figure 5. Heatmaps illustrating the predictive performance of different combinations of feature selection methods (rows) and classification algorithms (columns). (a-c) Cross-validated AUC values of models predict grade, Ki-67, and combined grade & Ki-67 based on T2-2D on the training set. (d-f) Cross-validated RSD values of models predict grade, Ki-67, and combined grade & Ki-67 based on T2-2D on the training set.


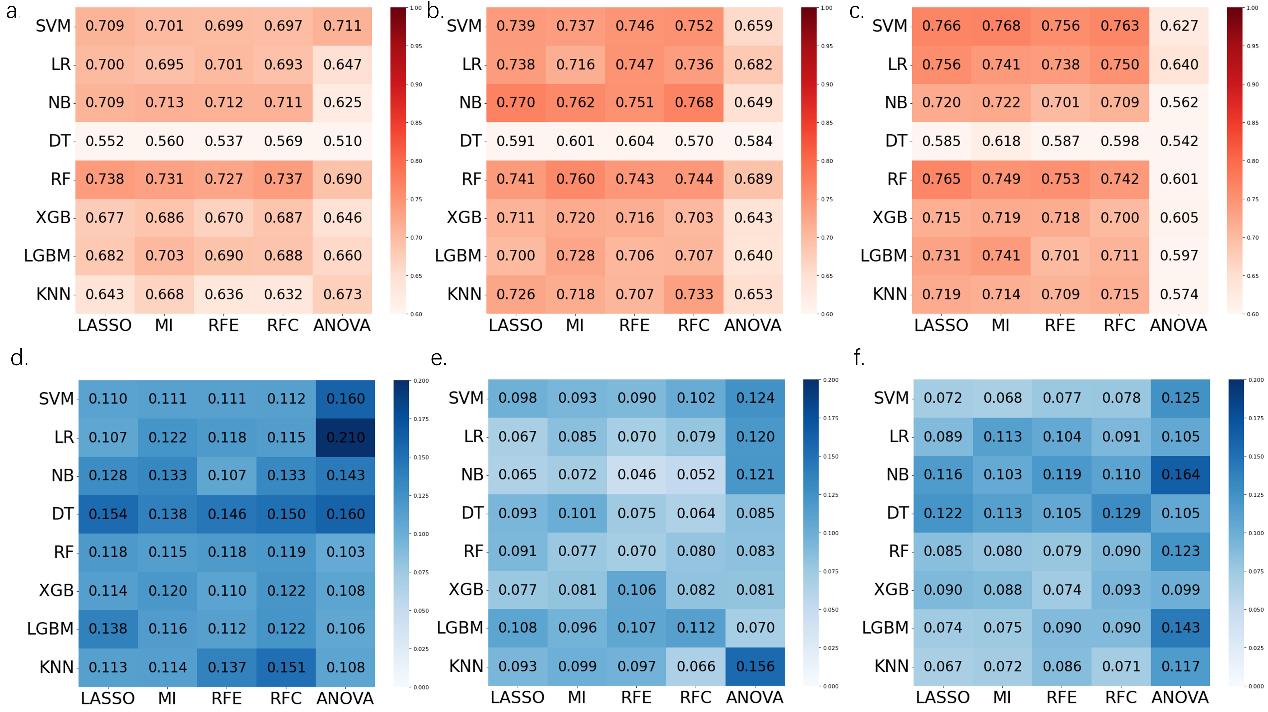


Supplementary Figure 6. Heatmaps illustrating the predictive performance of different combinations of feature selection methods (rows) and classification algorithms (columns). (a-c) Cross-validated AUC values of models predict grade, Ki-67, and combined grade & Ki-67 based on T2-3D on the training set. (d-f) Cross-validated RSD values of models predict grade, Ki-67, and combined grade & Ki-67 based on T2-3D on the training set.

**Software Packages Used:**

import pyradiomics

from sklearn.model_selection import train_test_split, KFold, StratifiedKFold, RepeatedKFold

from sklearn.linear_model import LogisticRegression

from sklearn.tree import DecisionTreeClassifier

from sklearn.svm import SVC

from sklearn.ensemble import RandomForestClassifier

from sklearn.naive_bayes import GaussianNB

from xgboost.sklearn import XGBClassifier

from lightgbm import LGBMClassifier

from sklearn.neural_network import MLPClassifier

from sklearn.neighbors import KNeighborsClassifier

from sklearn.metrics import f1_score, precision_score, recall_score, roc_auc_score, accuracy_score, roc_curve

from scipy.stats import ttest_ind, levene, stats

from sklearn.preprocessing import StandardScaler

from sklearn.feature_selection import SelectKBest

from sklearn.feature_selection import mutual_info_classif as MI

from sklearn.feature_selection import RFE

from sklearn.feature_selection import SelectFromModel

from sklearn.feature_selection import VarianceThreshold

from sklearn.utils import shuffle

from imblearn.over_sampling import SMOTE

from pymrmr import mRMR

from sklearn.model_selection import RepeatedStratifiedKFold

**Parameter of feature selection and machine learning model:**

SVC (probability=True, random_state=25, class_weight='balanced')

LogisticRegression(random_state=25,max_iter=100000,class_weight='balanced',solver='liblinear')

GaussianNB()

DecisionTreeClassifier(random_state=25, criterion='entropy')

RandomForestClassifier(criterion = 'entropy',random_state=25)

XGBClassifier(random_state=25)

lgb.LGBMClassifier(random_state=25)

KNeighborsClassifier()

**Detail of Image Acquisition**

In the center A, MRI was performed on a 3.0T Philips Achieva. The detailed protocols and parameters were as follows: Slice thickness was 5mm, with a repetition time of 2000ms and echo time of 20ms for T1-weighted imaging (T1WI), and a repetition time of 3000ms and echo time of 80ms for T2-weighted imaging (T2WI). For contrast-enhanced T1WI, the repetition time was 18ms and echo time was 4.6ms.

In the center B, MRI was performed on a 1.5T Siemens Avanto. The detailed protocols and parameters were as follows: Slice thickness was 5mm, with a repetition time of 1800ms and echo time of 6.8ms for T1WI, and a repetition time of 3500ms and echo time of 120ms for T2WI. For contrast-enhanced T1WI, the repetition time was 2000ms and echo time was 2.6ms.

In the center C, MRI was performed on a 1.5T GE Signa HDxt. The detailed protocols and parameters were as follows: Slice thickness was 5mm, with a repetition time of 1700ms and echo time of 20ms for T1WI, and a repetition time of 5000ms and echo time of 120ms for T2WI. For contrast-enhanced T1WI, the repetition time was 200ms and echo time was 2.49ms.

In the center D, MRI was performed on a 3.0T Siemens Skyra. The detailed protocols and parameters were as follows: Slice thickness was 5mm, with a repetition time of 487ms and echo time of 15ms for T1WI, and a repetition time of 4000ms and echo time of 110ms for T2WI. For contrast-enhanced T1WI, the repetition time was 542ms and echo time was 15ms.

**Parameter of Radiomics Feature Extractor:**

2D:

'minimumROIDimensions': 2, 'minimumROISize': None, 'normalize': True, 'normalizeScale': 100, 'removeOutliers': None, 'resampledPixelSpacing': [1, 1, 1], 'interpolator': 'sitkBSpline', 'preCrop': True, 'padDistance': 10, 'distances': [1], 'force2D': True, 'force2Ddimension': 0, 'resegmentRange': None, 'label': 1, 'additionalInfo': True, 'binWidth': 25}

features {'shape2D': None, 'firstorder': None, 'glcm': None, 'glrlm': None, 'glszm': None, 'gldm': None, 'ngtdm': None

3D:

'minimumROIDimensions': 2, 'minimumROISize': None, 'normalize': True, 'normalizeScale': 100, 'removeOutliers': None, 'resampledPixelSpacing': [1, 1, 1], 'interpolator': 'sitkBSpline', 'preCrop': False, 'padDistance': 5, 'distances': [1], 'force2D': False, 'force2Ddimension': 0, 'resegmentRange': None, 'label': 1, 'additionalInfo': True, 'correctMask': True, 'binWidth': 25}

features {'shape': None, 'firstorder': None, 'glcm': None, 'glrlm': None, 'glszm': None, 'gldm': None, 'ngtdm': None}
